# A Very Rare Abnormal Condition of the Iris

**Published:** 1873-06

**Authors:** E. L. Holmes

**Affiliations:** Chicago


ARTICLE III.—*A very Rare Abnormal Condition of the Iris.* By E. L. HOLMES, M.D., of Chicago.

J. T., æt. 23, student of medicine, consulted me last December regarding a singular affection of both eyes, which, he stated, his parents believed to be congenital. The patient was of light complexion and hair, and had always enjoyed good health.

I found clusters of peculiar jet black fungoid growths surrounding the whole pupillary border of each iris, and projecting two or three lines into the anterior chamber—similar to the growths—the corpora nigra—so often found on the pupillary edge of the iris in horses.

These little tumors, seven or eight in number, five of which were relatively large and three very small, were pedunculated, quite regular in form, and pear shaped. They seemed velvety but not glossy, and less granular than the "corpora nigra" in horses. The pupils, or rather apertures, in the centres of these clusters were very narrow and irregular.

At the distance of a few feet scarcely any abnormal appearance could be discovered. Strangers and even associates have seldom remarked the peculiarity. These growths, jet black on a very light blue iris, presented the appearance at a short distance of a normal pupil, which, however, was quite elliptical in each eye.

The iris lay in its normal plane and showed no indication of tumors situated behind it. In the left eye one of the smaller tumors, attached to the lower border of the pupil by a very minute hair-like peduncle, had free tremulous motions with every movement of the eye.

There was no perceptible contraction or dilatation of the pupil on exposure to the influence of light and shade, although a very slight spasmodic action of the iris was perceptible.

The patient had always supposed till recently that his vision was perfect except for distant objects. The myopia was greater during childhood than subsequently. During the past four years he has worn concave lenses No. 30, for distant objects, and has always been able to study and write constantly without difficulty.

Four months ago vision of the left eye began to fail; useful vision is now almost extinct. Six weeks ago the other eye began to fail. At present with the left eye the patient can read common print indistinctly for a few moments, when the letters become almost invisible.

By means of a stenopaic slit held in a proper position, or even a minute circular aperture in a disc, vision is much improved.

Four weeks ago vision was rendered almost perfect in the left eye and much improved in the right one, both for distant and near objects, by a concave cylindrical lens No. 20, so placed that the axis, passing from above downward and outward (in each eye) formed an angle of 45° with the perpendicular diameter of the cornea.

On account of the extreme minuteness of the pupils an ophthalmoscopic examination of the fundus was impossible, especially as I did not wish to try even so simple an experiment as dilating the pupils with atropine.

I advised iridectomy in the right eye, but have not heard from the patient since my last examination in February.

